# Being inspired: What we have learned about picky eating in childhood from using questionnaires on feeding practices and behaviors in a longitudinal birth cohort

**Published:** 2021

**Authors:** Pauline M. Emmett, Caroline M. Taylor

**Affiliations:** Centre for Academic Child Health, University of Bristol, 1-5 Whiteladies Rd, Bristol BS8 1NU, UK

## Abstract

We have made an extensive study of the development of picky eating behavior in childhood, and its effects on diet and growth, using data from the Avon Longitudinal Study of Parents and Children (ALSPAC). Questions, inspired by experience, were asked at regular intervals about difficulties parents had in feeding their child and how they responded to these difficulties. The data collected have provided insight into the development and consequences of being a picky eater in childhood. The importance of collecting prospective data on diet and feeding behaviors in early life studies is emphasized.

## Commentary

We have made an extensive study of the development of picky eating behavior in childhood, and its effects on diet and growth, using data from the Avon Longitudinal Study of Parents and Children (ALSPAC) [1]. Mothers were recruited during pregnancy in 1991/2 and they and their child have been followed using questionnaires plus measurements at research clinics from infancy onwards. A wealth of information was collected prospectively about feeding the child in their first 1000 days and beyond. Questions were asked at regular intervals about difficulties parents had in feeding their child and how they responded to these difficulties. Food frequency questionnaires (FFQ) about the child’s diet were completed by the main carer, usually the mother, and at certain ages records of all foods consumed by the child were collected in diet diaries. In this article we will describe how these data have provided us with an opportunity to gain insight into the development and consequences of a child being classified, retrospectively, as a picky eater. We will show the importance of collecting prospective data on diet and feeding behaviors in early life studies by summarizing our findings from the responses to the questionnaires.

From the instigation of ALSPAC, diet was considered to be a very important contributor to the health and wellbeing of children. In designing the questionnaires for the study Jean Golding, the founder of ALSPAC, and Pauline Emmett, the nutrition lead, were inspired by personal experience of parenting as well as training and observation. We recognized that some children become very choosy about foods and that some parents find this behavior difficult to manage. This led to the inclusion of questions asking about various feeding difficulties that might occur, and the regular incorporation of FFQ tailored to the age of the child. Table 1 lists the timing of questions sent to mothers in the study, in postal self-completion questionnaires, and gives some examples of the wording of the questions and answer categories.

The first analyses investigating the answers to the questions that we were inspired to include were on the age of introduction of lumps (chewy foods) to the diet of infants [2]. The recommended age for the introduction of lumpy foods was 6-9 months of age; this was achieved by most parent/infant pairs, but 11% had introduction before 6 months and 17% from 10 months (Table 1). There were some differences in foods and drinks fed to the infants at 6 and 15 months relating to the age of introduction, but the main difference was in the proportion of parents experiencing difficulty feeding the child [2]. If the child had refused to take solid foods at any point up to 6 months of age the likelihood of the parents introducing lumps late increased compared with introduction at the recommended age (OR 1.53 (95% CI 1.36, 1.73) in adjusted analysis). The particular aspect of feeding difficulty at 15 months most strongly associated with late introduction of lumpy foods was the child having definite food likes and dislikes (OR 1.91 (95% CI 1.68, 2.16) in adjusted analysis). We investigated food consumption and feeding difficulty outcomes again when the children were aged 7 years: there was continued evidence of choosiness in the children introduced late to lumpy foods (OR 1.15 (95% CI 1.05, 1.28) in adjusted analysis) [3]. Furthermore, these children were more likely to have fewer than one portion a day of fruits or vegetables (OR 1.33 (95% CI 1.14, 1.55); OR 1.21 (95% CI 1.04, 1.41), respectively, in adjusted analysis). These findings showed the importance of introducing lumpy foods in a timely manner during infancy and were influential in obtaining further funding for our work.

This initial work led us to look more closely at the questions we had designed on choosy eating behavior in children to determine if we had an acceptable classification of picky eating. We found that many studies had used a question similar to the one we had included about definite likes and dislikes for food in children to define picky eating [4]. The answer ‘Yes, very choosy’ was used to define a child who was ‘very’ picky. We had asked this question several times during childhood (Table 1) so were able to assess picky eating behavior at several ages, and these longitudinal data enabled further definitions of transient and persistent picky eating (very picky at several age points). These definitions were applied retrospectively so were not known to the parents at the time of completing the questionnaires. We used these data to investigate picky eating in combination with the wide range of data collected by ALSPAC in two ways: (1) assessing early feeding practices and behaviors as antecedents of picky eating; and (2) assessing the nutritional and growth outcomes of picky eating in the general population of children.

Underpinning this investigation was our design of a series of questions about particular feeding difficulties: refusal to eat the right foods, being choosy with foods, and not eating enough foods (all as defined by the mother). For each of these questions we also asked if the mother worried about this problem if it occurred (Table 1). We used our basic definition of picky eating behavior, as described above, and found that children classified as very picky eaters at 3 years were more likely to have been difficult to feed at 15 months than children who were not picky at 3 years. This was especially so if the mother indicated that she was worried about the child’s eating behavior at 15 months [5]. Children who refused food and those who were choosy about food at 15 months and if their mother was very worried about these problems had greater odds of being classified as very picky at 3 years (OR 3.14 (95% CI 1.43, 6.17) for food refusal and OR 7.41 (95% CI 3.69, 14.88) for choosiness, in adjusted analysis). Not eating enough food at 15 months was not independently associated. Being introduced to lumps late was the only feeding variable from the first year of life that was independently associated with increased odds of being very picky at 3 years (OR 1.43 (95% CI 1.16, 1.78)). We further investigated the factors that predicted worry in the mothers and found that it was much more likely in first-time mothers (OR 2.14 (95% CI 1.25, 3.68), and when the mother found the child difficult to feed before 6 months (OR 1.53 (95% CI 1.05, 2.24) or the child refused solid foods before 6 months (OR 1.92 (95% CI 1.33, 2.76) [6]. If she was worried, the mother was also more likely to introduce lumps late (OR 2.33 (95% CI 1.60, 3.39)). These findings suggest that support to mothers, especially first-time mothers, during the time of introducing complementary feeding would be beneficial.

In addition to the FFQ and questionnaire data, from time to time we also collected records of foods eaten by the children in 3-day diet diaries. These were completed by the parents on behalf of the child at 3.5 and 7.5 years and by the children with the help of the parents at 10 and 13 years of age. The diaries were accompanied by a short questionnaire that asked for extra details about foods and drinks taken daily. This questionnaire included, for example, the types and thickness of slices of bread and fat spread used, plus details of drinks such as milk, tea, coffee and soft drinks, and a record of the volumes of cups/mugs/flasks usually used. This could be completed at home and brought with the diary to a research clinic. Whenever possible, the diary was checked for completeness with the child and parents by a nutrition fieldworker. At age 10 and 13 years most of the children were seen by a fieldworker. The information collected by these means was used to improve the accuracy of the dietary assessment. The nutrient and food intakes estimated in these children compared closely to those published by the UK’s National Diet and Nutrition Survey, which is carried out regularly in a random sample of British children [7]. At each research clinic the standing height of the child was measured to the last complete millimeter using the Harpenden Stadiometer (Holtain Ltd, Crymych, UK) and weight was measured to the nearest 0.1 kg using the Tanita Body Fat Analyzer (Model TBF 305, Tanita, Tokyo, Japan).

The nutritional aspects of this investigation have been summarized [8]. We found that the dietary intakes of children with picky eating behavior were generally adequate in nutrients compared with reference nutrient intakes (RNI). Only four micronutrients showed important differences: zinc, retinol equivalents, iron and niacin. For example, more children who were persistently picky between 24 and 65 months had zinc intakes below the lower RNI (inadequate for most individuals) at age 7.5 years (9.2%) than children who were never picky (5.9%, p<0.05). However, compared with non-picky children, picky children tended to eat less meat, fruits and vegetables [9] and less fiber overall [10]. At age 7.5 years persistently, picky children ate on average 37% less meat, 48% less vegetables and 33% less fruit by weight than never picky children. We also found that there remained some differences in dietary intakes between these groups of children when they moved into adolescence [11]. At age 13 years picky children (classified at age 3 years) ate on average less meat (8% lower), vegetables (23%) and fruit (14%) than non-picky children. Parents should be supported in strategies to increase children’s intake of fruits and vegetables from an early age.

The children who were very picky weighed slightly less on average and were slightly shorter on average than children who were not picky, but both groups were above the 50th centile on the UK growth charts for weight and height between 7 and 17 years [12]. The UK growth charts are based on WHO Child Growth Standards which are used throughout the world; therefore, British children tend to have measurements above the 50th centile relative to whole world standards. On average the very picky children followed the 75th centile while the non-picky children followed the 80th centile. Slightly more of the picky children were thin and slightly fewer were overweight or obese than the non-picky children and being a picky eater was predictive of being thin. These growth patterns suggest that most picky children grow well, and this should reassure parents. Those picky eaters who show a tendency to be thin may benefit from being monitored by health professionals.

Throughout childhood we asked parents about other aspects of their feeding relationship with their children. Questions included how parents deal with food refusal, whether mealtimes are enjoyable or difficult, who the child eats with, how the child helps around food selection, purchase and preparation, what intentions and influences the mother has around feeding the family, and so on. A study using some of these variables investigated the fruit and vegetable intake of 7-year-old children using data from the diet diaries [13]. The strongest independent predictor of children’s fruit intake was parents having an intention to feed fresh fruit everyday (about 50 g/day more fruit); the child being choosy was a predicter of a lower intake. For vegetable eating the child being very choosy was the strongest independent predictor of intake (about 18 g/day less vegetables). The child eating a variety of foods and the parents having an intention to feed vegetables or salad every day predicted increased intake. We intend to investigate picky eating further using these variables to enhance understanding of this behavior in children and further develop evidenced-based advice for parents and health professionals.

The strengths of ALSPAC include: (1) a large amount of prospectively collected data that is readily accessible to researchers [14] with both parents and the study child themselves providing data throughout the child’s life; (2) the use of both questionnaires and hands-on research clinics with trained and monitored staff collecting standardized data; (3) the efforts made to maintain the cohort over 30 years allowing the assessment of later outcomes related to infant and childhood practices; (4) persistent efforts to obtain funding from all possible sources that were sometimes successful.

Attention was given to keeping the participants involved from the beginning, this included birthday cards for the children, newsletters for parents, local media stories and more recently the use of social media. Great efforts were made to provide a welcoming and child-friendly age-appropriate atmosphere in the research clinics. The feedback from the cohort participants is generally very positive and many of them have very much enjoyed being involved. Obtaining funding to recruit and follow the cohort was particularly difficult at first with small amounts of funding brought together to keep the study going. However, once the study was established, had published some top-quality papers, and had started to accumulate quality data major funders became involved and a firmer footing for funding was established. The breakthrough for the picky eating investigation came in 2010 when we obtained European Union funding through the 7th Framework programme in a consortium with other European countries in the Habeat study (https://habeat.eu/). This led to industry funding being obtained from Nestle Nutrition for a programme of work concentrating on picky eating. It had taken 20 years to obtain funding to investigate this subject thoroughly.

The limitations include: (1) that the cohort was recruited from one geographical area of England so the findings may not always be generalizable; (2) there has been loss to follow up and this is biased towards decreasing the cover of the less educated groups; (3) the feeding questions used were not validated against objective data. Since the start of ALSPAC validated scales have become available to measure items such as child eating behaviors and parental feeding practices [15–18]. In a new study it would be prudent to incorporate these.

In conclusion using our original questions, inspired by experience but not validated at the time, we have produced a body of work that has contributed to knowledge in the area of infant feeding and particularly the development and consequences of picky eating behavior in childhood in a general population. This work on picky eating has shown the importance of researchers being willing to use inspiration from life experience when setting up long-term research projects. It is not always possible to know exactly how data will be used in the future, so it is important to think laterally and not be confined to set hypotheses that limit ideas.

## Figures and Tables

**Table 1:** Questions asked to ALSPAC parents about feeding their child at different ages, with the age of the child when the questions were asked by questionnaire.

Questions as asked	Answer categories	Age of child (months)
1. Please indicate if your baby had any of the following feeding behaviors and when they occurred:		
a) Slow feeding	Yes 0-3 months/Yes 4-6 months No not at all	6
b) Choking	Yes 0-3 months/Yes 4-6 months No not at all	6
c) Taking only small quantities at each fed	Yes 0-3 months/Yes 4-6 months No not at all	6
2. Do you feel you have ever had any difficulty feeding your baby?	Yes, great/Yes, some difficulties No, no difficulties	6
3. Has your baby refused to take solids before 6 months of age?	Yes No	6
4. Babies first solid meals are usually a puree. When did your child first start having meals with lumps in?	Before 6 months Between 6 and 9 months 10 months or more	15
5. Do you feel that you have had any difficulty feeding your child in the past year?	Yes No	15, 24, 38
6. Does your child have definite likes and dislikes as far as food is concerned?^1^	No, will eat almost anything Yes, quite choosy Yes, very choosy	15, 24, 38, 54, 65, 77, 115
7. Has your child at any time:						
a) Refused to eat the right foods?	Yes, worried me greatly Yes, worried me a bit Yes, but did not worry me No, did not happen	15, 24, 38, 54, 65, 77, 81, 103, 115, 157
b) Been choosy with food?	Yes, worried me greatly Yes, worried me a bit Yes, but did not worry me No, did not happen	15, 24, 38, 54, 65, 77, 81, 103, 115, 157
c) Not eaten enough food?	Yes, worried me greatly Yes, worried me a bit Yes, but did not worry me No, did not happen	15, 24, 38, 54, 65, 77, 81, 103, 115, 157

1 This question was used to define picky eating at each age and longitudinally [4].
